# A natural barrier: tick‐repellent potential of a spruce‐derived volatile blend against *Hyalomma excavatum* and *Ixodes ricinus*

**DOI:** 10.1002/ps.70296

**Published:** 2025-10-16

**Authors:** Martyn J. Wood, Sare I. Yavaşoğlu, James C. Bull, Serkan Bakırcı, Kanagasooriyam Kanagachandran, Tariq M. Butt

**Affiliations:** ^1^ Department of Biosciences Swansea University Swansea UK; ^2^ Institute of Molecular Biology and Biotechnology Foundation for Research and Technology‐Hellas (IMBB‐FORTH) Heraklion Greece; ^3^ Department of Biology Aydın Adnan Menderes University Aydın Türkiye; ^4^ Department of Parasitology Aydin Adnan Menderes University Aydın Türkiye; ^5^ Rentokil Initial 1927 plc Crawley UK

**Keywords:** blend, *Hyalomma*, *Ixodes*, repellent, volatile organic compounds

## Abstract

*Ixodes ricinus* and *Hyalomma excavatum* are two widely dispersed vectors of pathogens, including those that cause Lyme disease and Crimean–Congo haemorrhagic fever in the human population. Recently developed, plant‐derived, mosquito‐repellent blends have shown promise against other vector clades, and this study assesses these blends as potential tick repellents. Blends of (+)‐borneol, bornyl acetate, eugenol, isoeugenol and camphor were assessed in two formats: blends 3 and 4. Ticks were assessed using the moving object bioassay (*Ixodes*) or a dual‐choice behavioural assay (*Hyalomma*). Both blends were compared against negative controls and four commercially available synthetic repellents: *N*,*N*‐diethyl‐3‐methylbenzamide (DEET), 2‐(2‐hydroxyethyl)‐1‐methylpropylstyrene 1‐piperidine carboxylate) (Picaridin), 3‐(*N*‐*n*‐butyl‐*N*‐acetyl)‐amino‐propionic acid ethyl ester (IR3535) and *p*‐menthane‐3,8‐diol (PMD). Results demonstrate the efficacies of blends 3 and 4; moreover, both were more effective than the commercial repellents (*P* < 0.05). Blend 3 was marginally more effective than blend 4, and differences in the repellent action were noted for each of the tick species, suggesting broad‐spectrum vector‐repellent activities, irrespective of life strategy. Overall, this work demonstrates the clear potential of blends 3 and 4 as tick repellents that offer an improved vector response over currently available commercial repellents. Furthermore, that the same repellent blends are capable of tick repellency in addition to mosquito repellency, offers the potential for widely dispersed usage across a range of integrated vector management strategies. © 2025 The Author(s). *Pest Management Science* published by John Wiley & Sons Ltd on behalf of Society of Chemical Industry.

## Graphical abstract

Both active and passive questing ticks, *Hyalomma excavatum* and *Ixodes ricinus*, were assessed for behavioural responses to two novel plant‐derived repellent volatile organic compound blends. Both tick species were repelled by the products, and both novo blends were significantly more repellent than currently available commercial products.

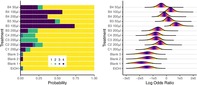

## 1 INTRODUCTION

Ticks are hematophagous ectoparasites of humans and animals with a worldwide distribution.^1^ They are vectors of broad range of pathogens, including protozoa, bacteria and viruses, that pose serious threats to human and animal health.^1^, ^2^ Tick‐associated economic impacts are significant on a global scale; in cattle alone, the impact of tick and tick‐borne diseases (TBDs) is estimated to range between US$14 and 19 billion.^3^ Lyme disease (*Borrelia burgdorferi*) infections account for an estimated 300 000–400 000 cases annually in the USA.^4^, ^5^ While incidence throughout Western Europe has been variable,^6^ the disease is considered endemic and of increasing concern with approximately 230 000 cases reported per year.^7^ Such incidence exerts a large burden on healthcare systems, causing significant associated costs in both human health and economic terms.

The sheep tick, *Ixodes ricinus*, is the most abundant tick in Europe,^8^ and is the vector of a wide range of pathogens of medical and veterinary importance including: *Borrelia burgdorferi, Anaplasma phagocytophilum*, *Babesia divergens*, tick‐borne encephalitis and Louping ill.^9^ Likewise, ticks belonging to the *Hyalomma* genus are common inhabitants of warmer Eurasian climates,^10^, ^11^ with *H. anatolicum, H. excavatum, H. marginatum* and *H. scupense* (syn *H. detritum*) particularly widely distributed.^12^, ^13^ *Hyalomma* species, including *H. excavatum*, also serve as vectors and reservoirs of important human and animal diseases including Crimean–Congo haemorrhagic fever, *Coxiella burnetii*, *Borrelia burgdorferi* s.s., *Rickettsia africae*, *R. aeschlimannii* and *Theleria annulata*.^14^, ^15^, ^16^, ^17^

Infection of humans and livestock with TBDs often requires intervention with therapeutics that are not only costly, but to which the responsible pathogens can develop resistance.^18^, ^19^, ^20^ In recent years, increases in tick activity and TBD incidence have been observed in many parts of the world, especially across the northern hemisphere where recording tends to be more common.^21^, ^22^, ^23^, ^24^ These increases are linked to many ecological and anthropogenic factors such as animal migration, the increased popularity of outdoor activities (e.g. walking, camping), changes in land usage (e.g. reforestation), increased trade and travel, socioeconomic downturns and climate change.^23^, ^25^, ^26^, ^27^, ^28^ Given the general increases in tick abundance, resistance and range, the potential for TBD proliferation is also rapidly increasing, driving a need for the development and adoption of effective prevention measures.

Two measures widely used to prevent the transmission of TBDs are the use of acaricides and repellents.^29^ Many chemical acaricides have been withdrawn or are restricted in use because of the risks they pose to human health and the environment.^30^, ^31^ Compounding these issues, widespread use of acaricides in domestic livestock has led to numerous reports of insecticide resistance in several ticks, particularly those belonging to Ixodidae.^32^ To mitigate these issues, much recent attention has focused on alternative methods of controlling ticks and TBDs, including the use of entomopathogenic fungi and plant‐based repellents and acaricides.^33^, ^34^, ^35^, ^36^, ^37^ Although direct control is often the focus, parallel use of repellents is also important in effective vector management because this can significantly reduce contact between ticks and their host organisms, thereby preventing attachment, and ultimately, disease transmission.

Ticks, like many other blood‐feeding arthropods, use olfaction to locate suitable vertebrate hosts for a blood meal. Ticks lack antennae but detect odours (e.g. carbon dioxide, ammonia, pheromones) using a structure on their forelegs called Haller's organ, which can also sense humidity, infrared light and heat.^38^, ^39^ To locate a host, ticks such as Ixodid species exhibit passive questing behaviours that involve the tick climbing up plant structures, waving their outstretched forelegs to detect host odours and then climbing onto a passing host.^40^

Tick repellents disrupt olfactory processes, ultimately reducing host‐seeking behaviour and subsequently bites. Most insect repellents also have tick‐repellent properties, including *N*,*N*‐diethyl‐3‐methylbenzamide (DEET), 3‐(*N*‐*n*‐butyl‐*N*‐acetyl)‐amino‐propionic acid ethyl ester (IR3535), 2‐(2‐hydroxyethyl)‐1‐methylpropylstyrene 1‐piperidine carboxylate (Picaridin), *p*‐menthane‐3,8‐diol (PMD) and citronella.^41^, ^42^, ^43^ Although extracts of many plant species have been shown to have tick‐repellent properties,^34^, ^44^ very few studies have identified the active compounds contained therein, and even fewer have screened individual plant‐derived compounds.^36^, ^45^ One of the more promising plant‐based repellents, in which the responsible semiochemical compounds have been identified, concerns the use of (+)‐Nootkatone. This sesquiterpene, originally identified in grapefruit extracts, has been found to possess effective pesticidal and repellent properties against a broad range of tick and mosquito species.^46^, ^47^, ^48^ Many other naturally derived compounds have been shown to repel ticks also, including phenylethyl alcohol, *β*‐citronellol, cinnamyl alcohol, geraniol, *α*‐pinene, carvacrol, thymol, nepetalactone, *p*‐anisaldehyde and eugenol.^49^, ^50^, ^51^, ^52^ Of these, a large number have been found to also repel hematophagous pests other than ticks, especially mosquitoes, highlighting the cross‐class efficacy of plant‐derived compounds and demonstrating their broad potential for biological control development.^53^, ^54^ However, the majority of research on these has focused on screening the individual compounds and not blended combinations, even though blends could enhance repellency through synergistic interaction. Furthermore, most studies have focused on extracts of angiosperms, not gymnosperms, as a source of repellent compounds. Recently, two blends of synergistic spruce‐derived volatile terpenes – borneol, bornyl acetate, eugenol, isoeugenol and camphor – were shown to be highly effective in repelling mosquitoes.^55^, ^56^ The aim of this study was to evaluate the repellent efficacy of these innovative blends against *I. ricinus* and *H. excavatum*, two disease vector species representing passive ‘sit and wait’^57^ and active chemotaxis^58^ questing strategies, respectively.

## 2 MATERIALS AND METHODS

### 2.1 Ticks

Assays were conducted using *I. ricinus* nymphs because this the most abundant developmental stage in the field and the most prevalent in disease transmission.^59^ Ticks in the field were collected via the dragging method in Gower, UK during summer 2018; the method predisposing collected ticks to demonstrating active questing behaviour. Collected ticks were separated by age and maintained in 30 × 30 × 15 cm plastic Tupperware boxes with moistened cotton wool to provide humidity at 21 ± 1 °C. Healthy *I. ricinus* nymphs were used for all *Ixodes* assays.

For assays focusing on *H. excavatum*, adult ticks were taken from the permanent laboratory‐maintained colony at the Department of Parasitology, Aydin Adnan Menderes University. This colony was established using engorged female ticks collected from naturally infested cattle in the Aydin region and subsequently reared *in vivo* using rabbits (New Zealand whites) and gerbils under the SOP and associated ethical guidelines set out at Aydin Adnan Menderes University (no: 64583101/2016/197). To prevent constant activation of their questing behaviour, ticks were kept in an incubator at 12 ± 1 °C and 85–90% relative humidity (RH) (Memmert IPP 110, Schwabach, Germany) until use. All individuals were activated by increasing the temperature of their enclosure to 27 ± 1 °C, 24 h before experimentation. Only those ticks responding positively towards human hand odours during handing were used for assay. All *H. excavatum* ticks used in trials were 1‐month‐old adults at the point of experimentation.

### 2.2 Repellent blends

The two repellent blends, referred to as blends 3 and 4 as in earlier work, were prepared as outlined previously.^56^ Blend 3 consisted of (75 mg) borneol, (75 mg) bornyl acetate, (75 μL) eugenol and (75 μL) isoeugenol. Blend 4 contained the same components as blend 3 with the addition of (75 mg) camphor. Both blends were diluted in pure ethanol (1:1) and three different volumes (50, 100 and 200 μL) were assayed in each experiment. All chemicals were purchased from Sigma‐Aldrich unless indicated otherwise and generally had >98% purity.

In *I. ricinus* assessments, the blends were compared with negative controls (no treatment or ethanol diluent only) and several commercial repellents. The commercial products tested included DEET (Anti‐Brumm® Forte 30% w/w, Hermes Arzneimittel GmbH, Pullach i. Isartal, Germany), Picaridin (Autan® Protection Plus 20% w/w, SC Johnson GmbH, Ekrath, Germany), PMD (Anti‐Brumm® Naturel, 20% w/w, Hermes Arzneimittel GmbH, Pullach i. Isartal, Germany), and IR3535 (Jaico Muggenmelk Natural, 19.6% w/w, Omega pharma Nederland BV, Rotterdam, The Netherlands).

In *H. excavatum* experiments, the same blend 3 and 4 preparations were compared with negative controls consisting of both no treatment (free filter paper) and ethanol‐impregnated filter paper, and a positive control consisting of commercial repellent. The commercial repellent used as positive control was PMD (Incognito, Holland & Barrett, UK) because this was found to be the most effective commercial preparation in the earlier *Ixodes* experiments. Methodological approaches to the deployment of the repellents (Section 2.3 and Section 2.4) were varied according to the different questing behaviours of each tick species.

### 2.3 Evaluation of repellent blends against *Ixodes ricinus*

**Figure 1:**
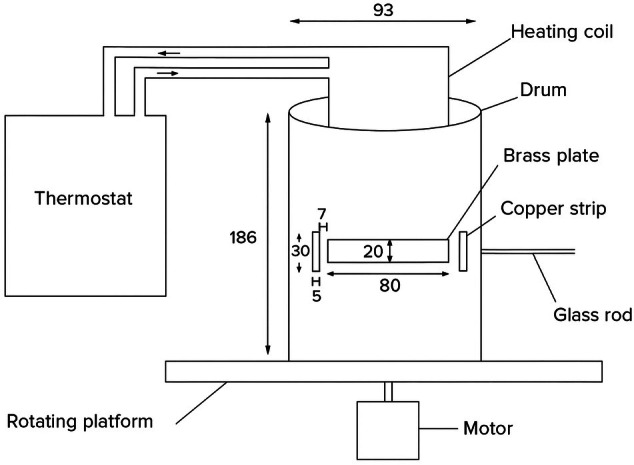
Depiction of the moving object assay set‐up. Figure adapted from Dautel *et al*.^60^

Tests were conducted according to the moving object bioassay (MOB) suitable for ‘sit and wait’ questing behaviours, as described by Dautel *et al*. The assays were conducted by Biogents (Regensberg, Germany) using their proprietary apparatus. This bioassay yields highly reliable, reproducible results similar to tests involving human volunteers.^60^, ^61^ In the MOB, warmth and motion are used as attractants, stimulating the natural tick behaviour of clinging to a passing host under controlled laboratory conditions. A slowly rotating vertical drum is heated to body temperature (35–37 °C) with the temperature being carefully monitored using a remote infrared thermometer. A piece of filter paper (5 × 10 cm) is fixed at an elevated position from the drum, and serves as the tick attachment site (Fig. 1). Ticks attracted to the heat gradient approach the drum on a horizontally positioned glass rod that ends directly in front of the drum at a distance at which the tick cannot reach the drum surface with its forelegs. As the drum rotates, however, the elevated surface of the drum covered with filter paper passes periodically, and the tick is able to latch on to and transfer to the drum.

To test for repellency, the novel repellent blends were applied to the filter paper at doses of 50, 100 and 200 μL, corresponding to 1, 2 and 4 μL cm^−2^, respectively. The highest of these doses has been found to work effectively against mosquito species^55^, ^56^; however, for Culicoides midges only lower dosages worked well (unpublished data). As such, a range of doses was included in the tick study to ensure that there was no over‐ or under‐stimulation of the ticks that might affect response. The commercial repellents and ethanol control were tested at the highest doses only (200 μL), well within manufacturer‐recommended efficacy ranges. The filter paper was replaced between each treatment. After each of the repellents was added to the filter paper, the filter papers were allowed to dry for 1 min before the beginning of the assay; in each replicate the repellents were applied to the filter paper immediately prior to experimentation. Observations on tick approach and drum transfer were recorded as measurements in time, alongside additional recordings relating to the duration of the tick's stay on the filter paper. Tick behaviour was measured at each step of the experimental process to reveal any subtle repellent effects. Behaviour observations were analysed using the Observer® software package (Nodus Information Technology, Wageningen, The Netherlands). Nymphs were gently placed at a line marked on the glass rod, ~8–12 mm from the tip. Standard experiments were performed at 21 ± 1 °C and 40–70% RH. Experimenters maintained a reasonable distance from the equipment during test runs to avoid the risk of human volatile emanations interfering with results. Ten nymphs were used for each treatment and the whole study repeated in triplicate. Tick behaviour was observed for 120 s in each replicate assay.

### 2.4 Evaluation of repellent blends against *Hyalomma excavatum*

**Figure 2:**
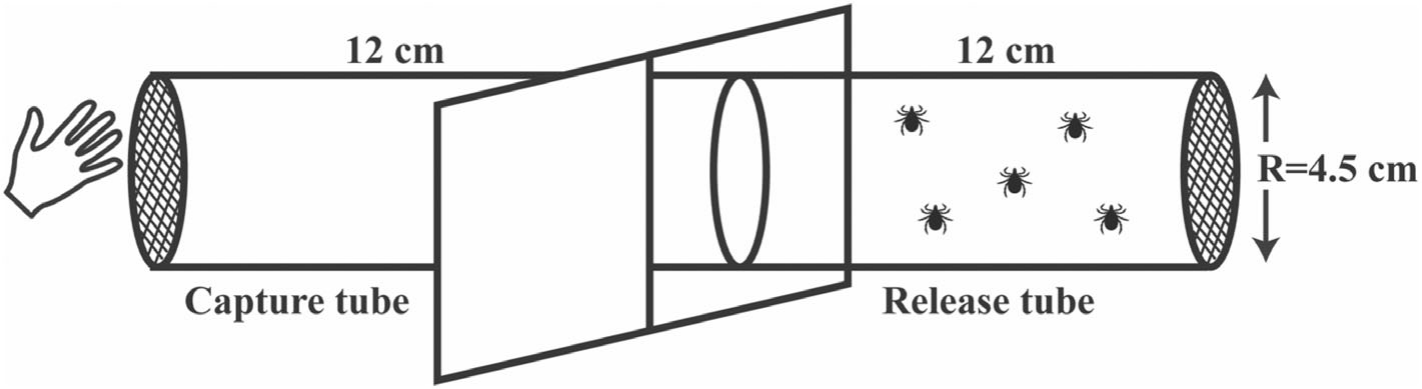
Representative diagram of the repellency test system for *Hyalomma excavatum* adults.

Because of the highly active questing nature of *Hyalomma* ticks, whereby orientation can take place from distances of 50–500 m,^62^ a new methodological approach approximating natural behavioural responses was required. Cylindrical plastic tubes (length = 12 cm, radius = 4.5 cm) developed for the World Health Organization's standard insecticide susceptibility tests were used.^63^ Two tubes, interconnected lengthways (Fig. 2), were capped with a mesh gauze and screw‐cap was placed at the outer ends of each tube to prevent ticks from exiting. One tube was designated a ‘release tube’, from which the ticks were released into the test environment. The other, designated the ‘capture tube’ in this experiment, was used to assess the behavioural response of the ticks to human odours in the presence of the putative repellent treatments; the open end of the capture tube allowed for placement of the volunteer's hand as an attractant source. Although no contact was made at any point between the human volunteers and the ticks, the full procedure was outlined to volunteers before the assay, and informed consent obtained from each volunteer. Filter papers impregnated with repellent treatments or control were placed at the junction of the two tubes, leaving a 12 cm approach to the repellent and a further 12 cm distance between the treatments and the human odour source. As with the *Ixodes* trials, repellents were applied to the filter papers at 50, 100 and 200 μL, corresponding to 1, 2 and 4 μL cm^−2^, respectively. Ticks were assessed individually, with four males and three females used per replicate set; trials for each treatment were repeated twice (*n* = 14). Tick behaviour was recorded for a total of 15 min with the two measured parameters being: (i) the length of time that each tick took to reach the treated filter paper, and (ii) the length of time that the ticks took to reach the human odour source. Ticks that failed to reach these setpoints within the timeframe were designated as taking ꝏ minutes for the purposes of analysis. All experiments were conducted at 21 ± 1 °C and a total of seven ticks (four male, three female) were released from the release tube. Tick behaviour was observed for 15 min, recording: (i) tick numbers that arrived at the filter paper and the time taken for this; and (ii) tick numbers that reached to the volunteer's hand and the time taken for this.

After every repellent test, the cylindrical tubes were cleaned with ethanol to avoid any repellent effect. Standard experiments were performed at room temperature 21 ± 1 °C and 40–70% RH.

### 2.5 Statistical analyses

Times taken to ‘tip of rod’, for ‘transfer to drum’ and ‘on drum’ (*Ixodes* trials) were right‐censored at 120 s, whereas ‘time to filter’ and ‘time to hand’ (*Hyalomma* trials) were right‐censored at 15 min. Therefore, the effects of blends and controls on these were modelled using Tobit analysis with log‐normal errors. Pairwise multiple comparisons between modelled estimates for all blends were made using Tukey familywise error rates.

Additional behavioural data were collected, categorizing individual tick outcomes in increasing severity of repellence as the tick: (i) ‘did not fall off the paper on the drum’, (ii) ‘fell off the paper’, (iii) ‘fell off tip of the rod while contacting the paper’, and (iv) ‘fell off tip without contacting the paper’. These outcomes were treated as ordinal categorical data and the effects of different blends and controls on the frequency of these behavioural outcomes were modelled using (Generalised Linear Mixed Models) GLMMs, with logit link functions. To handle the experimental design of trials as a random effect, modelling was undertaken in a Bayesian framework, with estimated log odds ratios described by their posterior distributions.

All statistical analysis was conducted using R version 4.05 (R Core Team, 2020). Tobit analysis was conducted using the package censReg.^64^ Ordinal regression was conducted using the package MCMCglmm.^65^ Pairwise multiple comparisons were produced using the package multcomp.^66^

## 3 RESULTS

### 3.1 *Ixodes ricinus* time trials

#### 3.1.1 Time to tip of the rod

**Figure 3:**
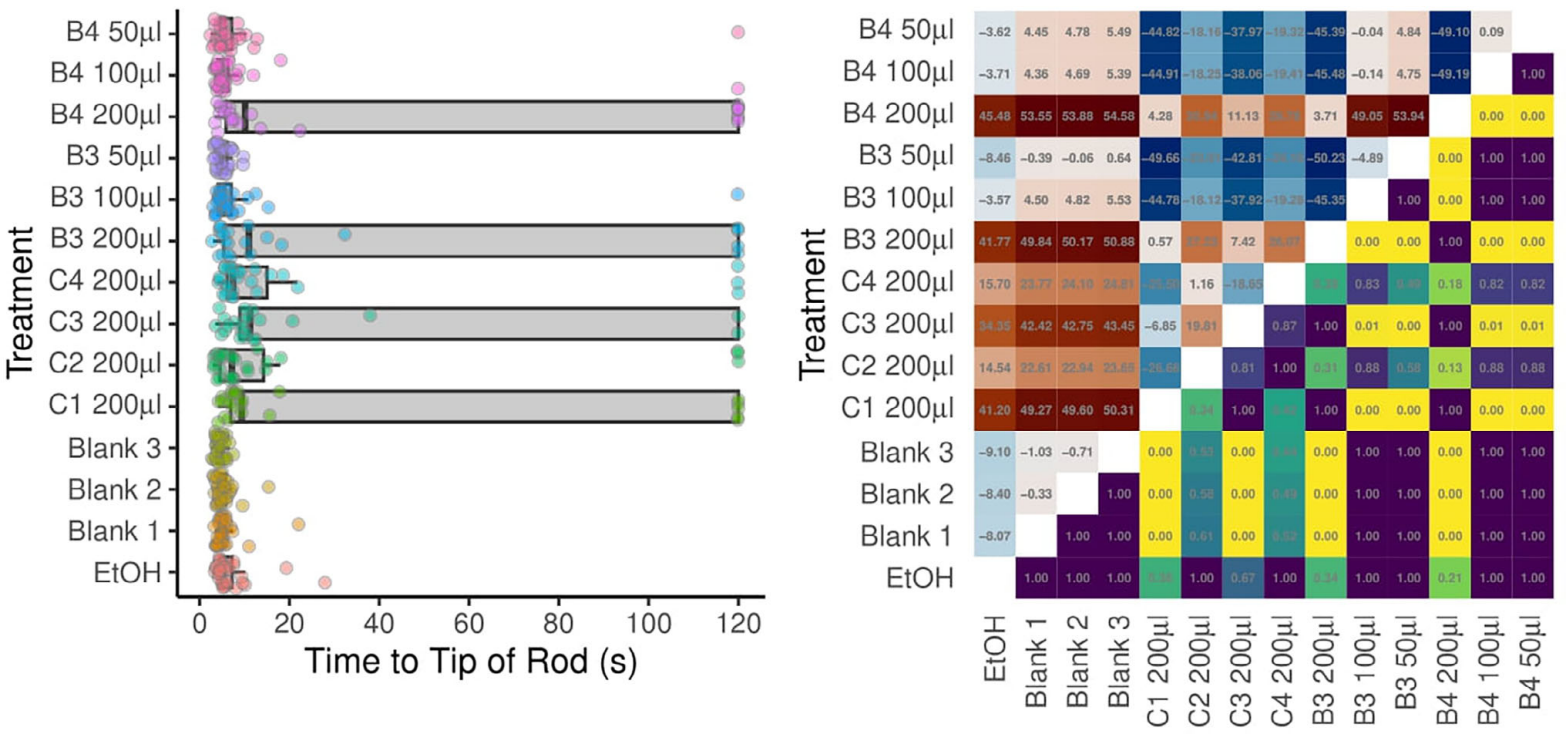
Graphical depictions of the time taken for *Ixodes ricinus* ticks (*n* = 30 per treatment) to reach the tip of the rod in the moving object assays designed to assess the repellent efficacy of a range of commercial and prototype repellent products. (Left) Box–whisker plots of times taken for ticks to reach the tip of the rod. The box midlines show median times, boxes span interquartile ranges (IQRs) and whiskers extend to an additional 1.5× IQR. Individual tick responses are shown as points with a small amount of vertical ‘jitter’ to prevent some points obscuring others. (Right) Pairwise comparisons between modelled response times. The upper triangle shows estimates of pairwise differences. The lower triangle of the matrix shows *P* values adjusted for multiple comparisons. For example, comparing between blend C1 200 μL and the ethanol control, the ticks appeared to take longer [estimate 41.2 s; row 5 from bottom, column (i)], but the difference was not statistically significant (*P* = 0.36; row 1, column 5). Commercial blends: C1, Anti‐Brumm Forte 200 μL; C2, Anti‐Brumm Naturel 200 μL; C3, Autan Protection Plus 200 μL; and C4, IR3535 19.6% ethanolic 200 μL. B3, blend 3; B4, blend 4 at various doses. Ticks indicated at 120 s were censored in the analysis.

At the 200‐μL dose, ticks presented with commercial repellent, as well as blend 3 or blend 4, took substantially longer to reach the tip of the rod than controls (Fig. 3). In all those cases, several ticks did not reach the drum within the 120 s observation period. Lower doses did not result in ticks taking statistically significantly to reach the tip of the rod compared with controls. Pairwise statistical comparisons between individual blends and controls are shown in Fig. 3.

#### 3.1.2 Time for transfer to the drum

**Figure 4:**
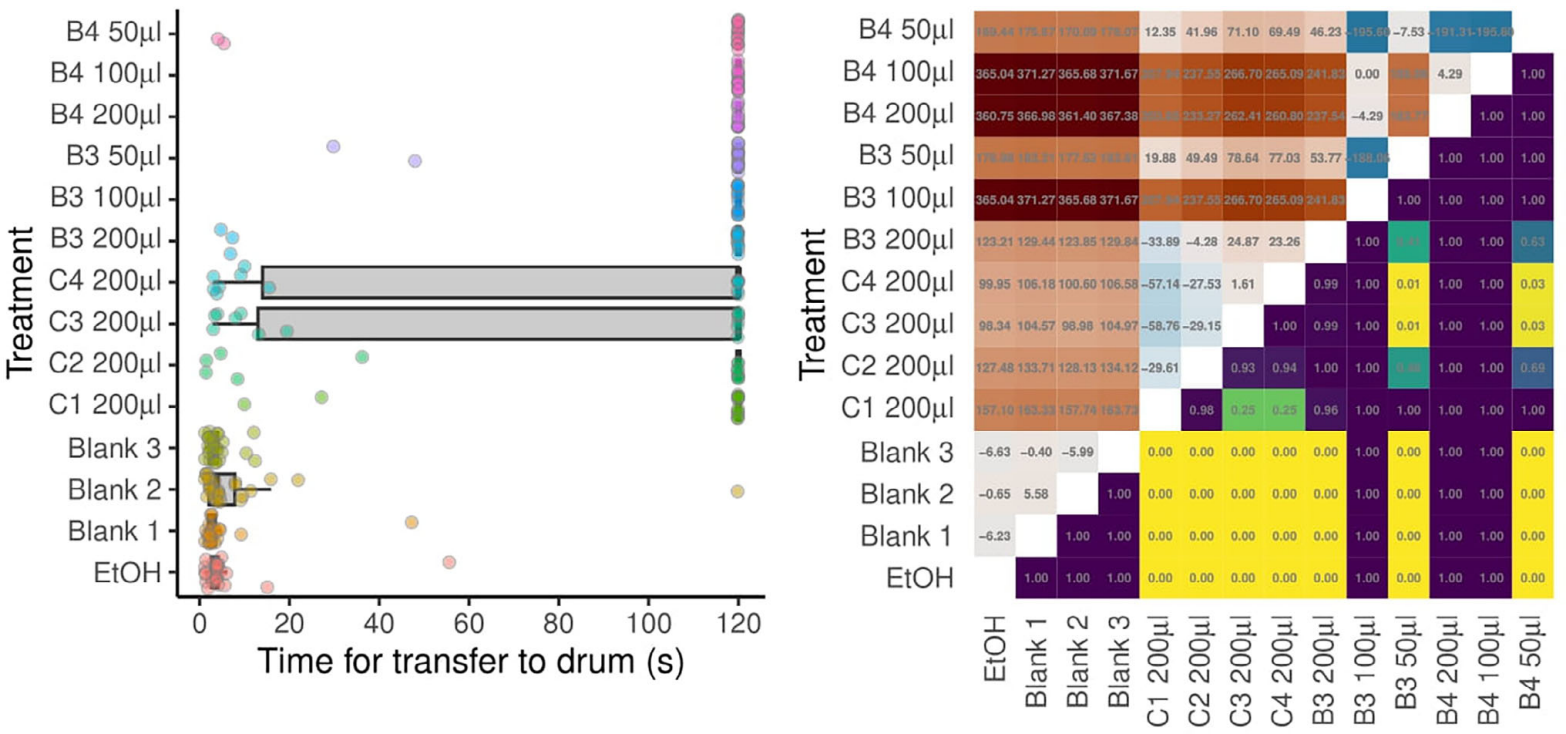
Graphical depictions of the time taken for *Ixodes ricinus* ticks (*n* = 30 per treatment) to transfer to the drum in the moving object assays designed to assess repellent efficacy of a range of commercial and prototype repellent products. (Left) Box–whisker plots of times taken for ticks to transfer to the drum. Box midlines show median times, boxes span interquartile ranges (IQRs) and whiskers extend to an additional 1.5× IQR. Individual tick responses are shown as points with a small amount of horizontal ‘jitter’ to prevent some points obscuring others. (Right) Pairwise comparisons between modelled response times. The upper triangle shows estimates of pairwise differences. The lower triangle of the matrix shows *P* values adjusted for multiple comparisons. Commercial blends: C1, Anti‐Brumm Forte 200 μL; C2, Anti‐Brumm Naturel 200 μL; C3, Autan Protection Plus 200 μL; and C4, IR3535 19.6% ethanolic 200 μL. B3, blend 3; B4, blend 4 at various doses. Ticks indicated at 120 s were censored in the analysis.

Ticks exposed to blend 3 at 100 μL and blend 4 at either 100 or 200 μL took longer than the observation period to transfer to the drum, if they did so at all, and were censored at 120 s in the analysis. However, because fewer of these ticks reached the tip of the rod, the resulting small sample size resulted in these differences not being statistically significant (Fig. 4). In all other cases/doses, all the repellents were associated with statistically significantly longer times for ticks to transfer to the drum. Pairwise statistical comparisons between individual blends and controls are shown in Fig. 4.

#### 3.1.3 Time on drum

**Figure 5:**
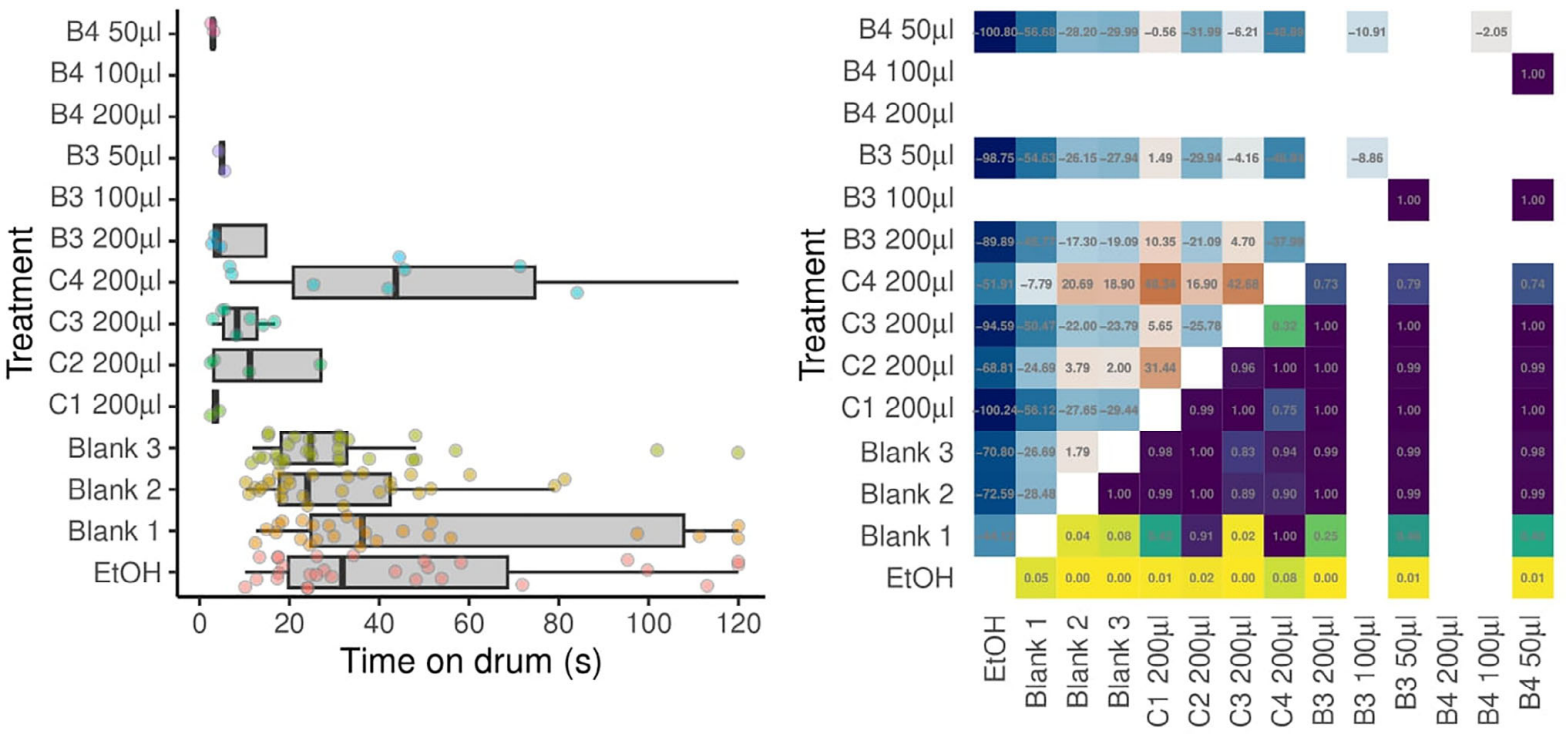
Graphical depictions of the time *Ixodes ricinus* ticks (*n* = 30 per treatment) spent on the drum in the moving object assays designed to assess repellent efficacy of a range of commercial and prototype repellent products. (Left) Box–whisker plots of times spent on the drum. Box midlines show median times, boxes span interquartile ranges (IQRs) and whiskers extend to an additional 1.5× IQR. Individual tick responses are shown as points with a small amount of horizontal ‘jitter’ to prevent some points obscuring others. (Right) Pairwise comparisons between modelled response times. The upper triangle shows estimates of pairwise differences. The lower triangle of the matrix shows *P* values adjusted for multiple comparisons. Commercial blends: C1, Anti‐Brumm Forte 200 μL; C2, Anti‐Brumm Naturel 200 μL; C3, Autan Protection Plus 200 μL; and C4, IR3535 19.6% ethanolic 200 μL. B3, blend 3; B4, blend 4 at various doses. Blend 3 at 100 μL, as well as blend 4 at 100 and 200 μL did not transfer to the drum, so pairwise comparisons were not made. Ticks indicated at 120 s were censored in the analysis. Missing treatments are those where no ticks transferred to the drum.

Ticks exposed to blend 3 at 100 μL and blend 4 at either 100 or 200 μL did not transfer to the drum, so no on‐drum times were recorded for these responses. Overall, ticks exposed to other blends spent less time on the drum than ethanol controls (Fig. 5); however, after multiple comparison testing, no significant differences in times spent on the drum were found between the blends and blank controls. Pairwise statistical comparisons between individual blends and controls are shown in Fig. 5.

#### 3.1.4 *Ixodes ricinus* behavioural trials

**Figure 6:**
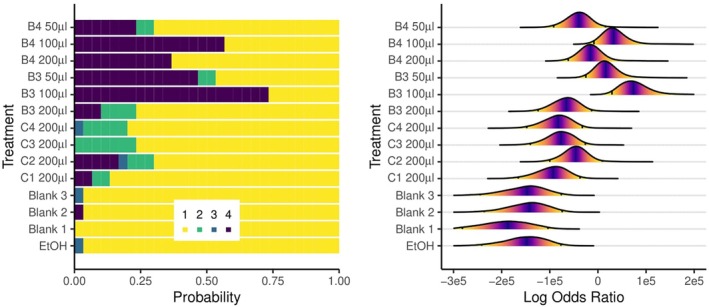
Graphical depiction of the relative probability of repellent behavioural effects elicited by repellent and control treatments by each of the commercial repellent products and the prototype repellent blends 3 and 4. (Left) Horizontally stacked bar charts of the probability of each tick behaviour under different blends and controls. Ordinal outcomes were the tick: (i) ‘did not fall off the paper on the drum’, (ii) ‘fell off the paper’, (iii) ‘fell off tip of the rod while contacting the paper’, and (iv) ‘fell off tip without contacting the paper’. (Right) Posterior distributions of modelled log odds ratios between behavioural outcomes. Higher log odds values indicate a higher probability of a more severe repellent behaviour associated with a particular blend or control. Vertical lines shown within distributions are 95% credible intervals, with statistical significance indicated where 95% intervals do not overlap between treatments. Commercial blends: C1, Anti‐Brumm Forte 200 μL; C2, Anti‐Brumm Naturel 200 μL; C3, Autan Protection Plus 200 μL; and C4, IR3535 19.6% ethanolic 200 μL. B3, blend 3; B4, blend 4 at various doses.

Clear differences were observed in tick behavioural response to the blends compared with controls (Fig. 6). Very few control ticks were classified as having been repelled to any degree; the substantial majority reached the filter paper on the drum and remained there for the duration of the experiment. Commercial products elicited some form of behavioural response approximately 25% of the time, but this was most often categorized as ‘mild’ repellence; ticks were not being deterred from drum transfer but were seen to fall off afterwards (Fig. 6, left). Blends 3 and 4 elicited a much stronger repellence factor, with up to 50–75% of ticks falling off the tip of the rod without contacting the filter paper on the drum at the 100 μL dose. Overall, modelled log odds ratios indicated that commercial products did not show a statistically significant repellent effect, whereas blends 3 and 4 did, compared with the controls (Fig. 6, right).

### 3.2 *Hyalomma excavatum* time trials

#### 3.2.1 Time taken to reach the treated filter paper

**Figure 7:**
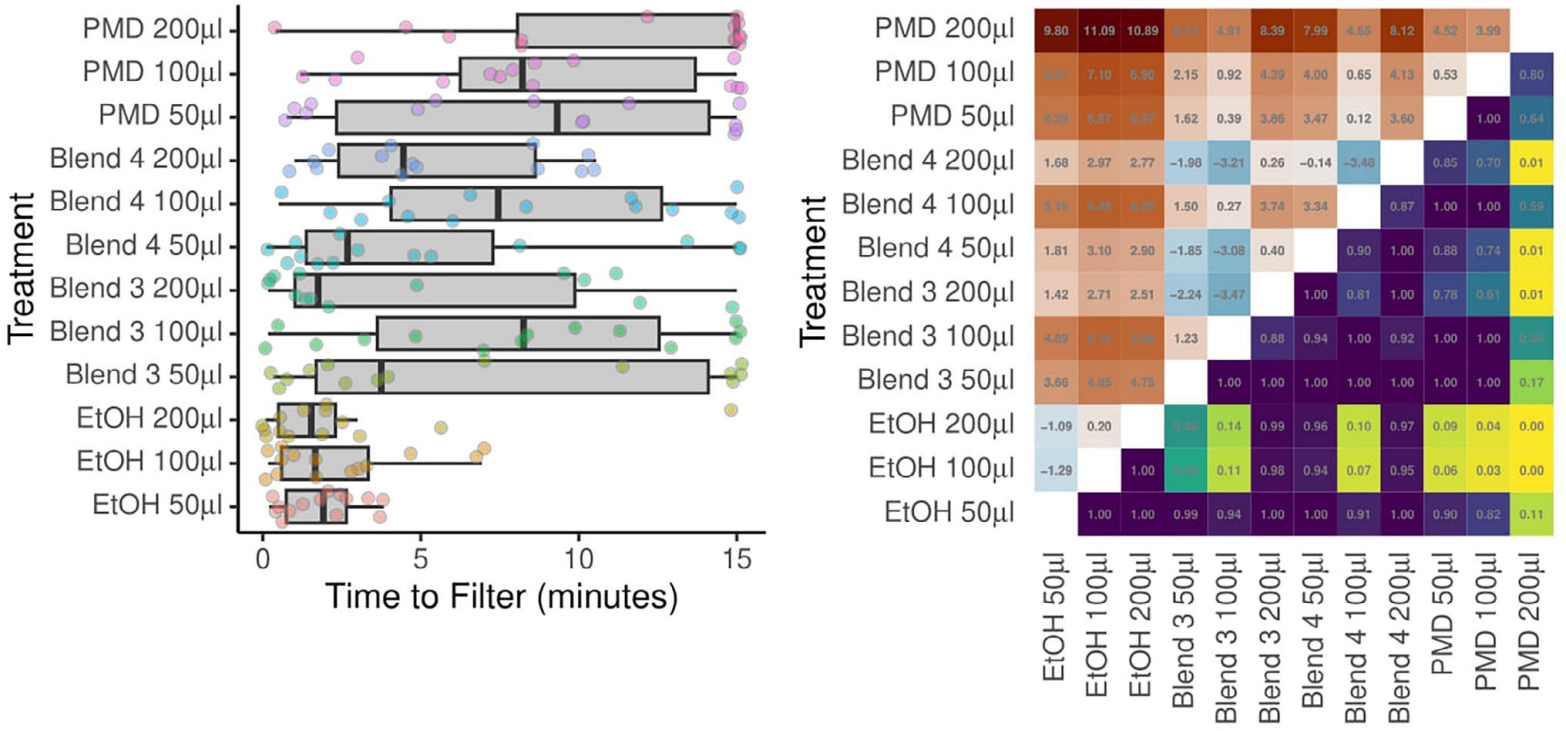
Graphical depiction of the time taken for *Hyalomma excavatum* ticks (*n* = 14 per treatment) to reach the treated filter papers in the tube‐repellency assays. (Left) Box–whisker plots of time taken to filter. Box midlines show median times, boxes span interquartile ranges (IQRs) and whiskers extend to an additional 1.5× IQR. Individual tick responses are shown as points with a small amount of vertical ‘jitter’ to prevent some points obscuring others. (Right) Pairwise comparisons between modelled response times. The upper triangle shows estimates of pairwise differences. The lower triangle of the matrix shows *P* values adjusted for multiple comparisons. Observations were curtailed (right‐censored) at 15 min.

There was little consistent evidence that the times taken to reach filters with repellents applied statistically significantly different from those with ethanol, with the exception of PMD at the 200 μL dose (difference compared with ethanol = 10.89 s, *P* < 0.0001) (Fig. 7). No significant differences were found between ethanol‐treated negative controls or blank controls with no treatment and all ticks were found to reach the filter paper rapidly.

#### 3.2.2 Times taken for *Hyalomma excavatum* ticks to reach human odour source

**Figure 8:**
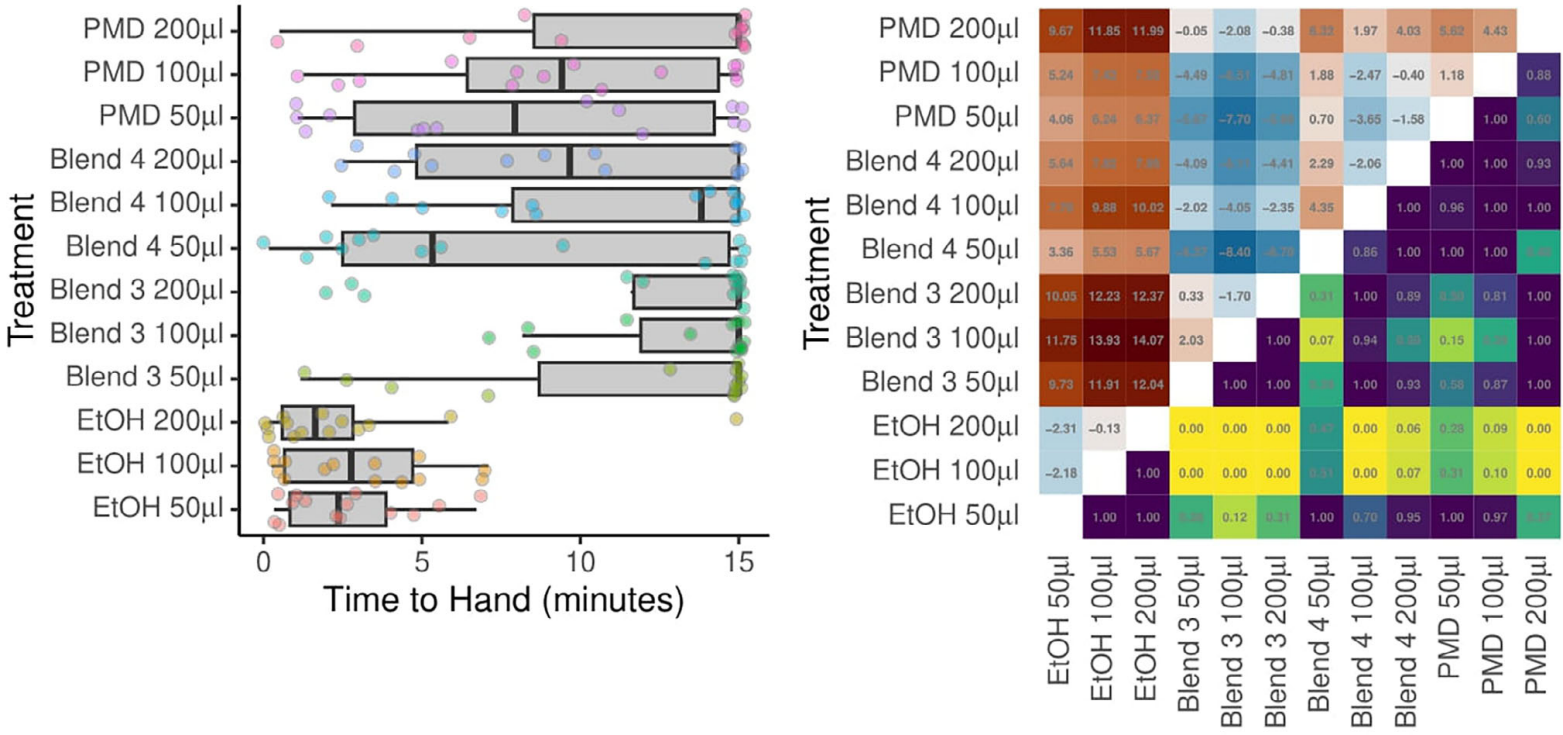
Graphical depiction of the time taken for *Hyalomma excavatum* ticks (*n* = 14 per treatment) to reach the human odour source present in the tube‐repellency assays. (Left) Box–whisker plots of time taken to test subject's hand. Box midlines show median times, boxes span interquartile ranges (IQRs) and whiskers extend to an additional 1.5× IQR. Individual tick responses are shown as points with a small amount of vertical ‘jitter’ to prevent some points obscuring others. (Right) Pairwise comparisons between modelled response times. The upper triangle shows estimates of pairwise differences. The lower triangle of the matrix shows *P* values adjusted for multiple comparisons. Observations were curtailed (right‐censored) at 15 min.

In contrast to the findings for movement to filters, times taken for ticks to reach the hand of the volunteer increased substantially when commercial repellent, as well as blend 3 or 4, were applied. The longest times were associated with blend 3 (difference compared with ethanol = 14.07 s at 200 μL, *P* < 0.0001), and this did not differ significantly with dose, whereas blend 4 was comparable to commercial repellent (Fig. 8). No significant differences were found between ethanol‐treated controls and untreated filter papers, all ticks reached the human odour sources rapidly.

## 4 DISCUSSION

This study demonstrates that blends 3 and 4, based on natural spruce compounds, were highly repellent to both *I. ricinus* and *H. excavatum* ticks, with blend 3 equally effective or more effective than any of the commercial repellents tested. Both blends have previously been shown to be highly efficacious repellents of adult mosquitoes of *Aedes*, *Anopheles* and *Culex*,^55^, ^56^ and have now also demonstrated significant repellent capability for ticks; broadening the spectrum of potential applications for the same formulations.

In the MOB targeting the ‘sit and wait’ *I. ricinus* nymphs, at the highest dose, both blends appeared to arrest tick movement and force retreat, suggesting that the volatiles were detected at some distance from the drum, thereby inferring spatial repellency. These effects appear similar to, and perhaps more effective than, the weak spatial repellency seen against mosquitoes.^55^, ^56^ Of those ticks that moved onto the drum, most soon fell off, independent of blend and dosage, suggesting either mild contact activity or disruption of tick sensory perception. Similar contact effects have been noted in *I. scapularis* exposed to a range of 20 plant‐derived essential oils,^67^ many of which also repel mosquitoes. Such results suggest that the blends may combine spatial repellency with contact effects, and in some cases may overpower tick olfactory apparatus. For example, at the highest dose of blend 3, ticks that transferred to the drum remained there for slightly longer than with commercial repellents, except IR3535, consistent with a temporary interference with sensory function.

In both *Ixodes* and *Hyalomma* behavioural assays, blend 3 appeared to be slightly more repellent than blend 4, dependent on the dose. The addition of camphor to blend 4, a well‐known repellent compound with acaricidal and insecticidal properties,^68^, ^69^, ^70^ appeared to have a negligible effect on the overall repellence of the blend against *I. ricinus*, and in the case of the *H. excavatum* appeared to reduce repellent efficacy somewhat. The same trends were noted previously,^55^ where the addition of camphor consistently reduced repellency against mosquitoes; however, this appears to be variable according to species. The other actives, borneol, bornyl acetate, eugenol and isoeugenol have previously been shown to possess insect‐repellent, insecticidal and ovicidal properties.^71^, ^72^, ^73^ Eugenol, and to a lesser extent isoeugenol, have been shown to possess acaricidal potential against scabies mites, *Sarcoptes scabiei*,^74^ whereas borneol and bornyl acetate had no effect against the two spotted spider mite *Tetranychus urticae*.^75^ However, little else is known about the tick‐repellent or acaricidal properties of these individual compounds.

Only a tiny percentage of the ticks transferred to the rotating drum in the presence of blends 3 and 4, and those individuals that did transfer quickly fell off, suggesting additional contact repellency or an overpowering of olfactory receptors by the repellent fumes. This pattern was also observed with the commercial repellents, demonstrating that individual ticks may have differentiated tolerances for the repellent compounds. Some researchers have hypothesized that putative repellents might induce an inhibition in the tick olfactory system, consistently reducing the ability to detect chemical volatiles and thus acting as deterrents.^76^, ^77^ Indeed, it has been proposed that DEET's mode of action is as an inhibitor, interfering with the recognition of attractant odours, rather than a true repellent, or as a modulator of the general olfactory receptor activity.^78^ However, given that ticks that successfully mounted the drum quickly fell off, it may be that contact repellency – noted for other plant compounds^67^ – takes effect, thereby mitigating any problems associated with habituation or olfactory inhibition in the ticks. Future work should seek to determine and address the modes of action through which botanical repellents work.

The actives in the current study – borneol, bornyl acetate, eugenol, isoeugenol and camphor – are present in spruce and many other plant species. Some of these compounds are present in plants or essential plant oils with known tick‐repellent properties such as *Rosmarinus officinalis* (rosemary), *Mentha spicata* (spearmint), *Origanum majorana* (marjoram), *Ocimum basilicum* (basil) and *Myristica fragrans* (nutmeg).^79^, ^80^ Although many compounds have been identified that repel ticks, to date, very few studies have evaluated them as blended actives. This study has demonstrated the efficacy of the novel mosquito‐repellent blends presented in Wood *et al*.^55^ against two of the most common tick species in European and Eurasian regions. One of the more interesting responses was that to DEET, in which ticks were found to be very inconsistently repelled. Although DEET remains one of the most utilized standard repellents, the literature suggests that such variability in response is not unheard of, especially in *Ixodes* ticks, which have shown highly variable responses across a few studies.^81^, ^82^, ^83^ In comparison with other natural repellents, the results are promising. PMD, originally isolated from lemon eucalyptus, is one of the few commercialized botanical repellents. In *Ixodes* experiments, PMD was found to be the most effective of the commercial repellents; however, spruce‐derived blends 3 and 4 were more effective than even PMD. That the spruce‐derived blends consistently repelled ticks compared with the industry standard synthetic and natural repellents, DEET and PMD, demonstrates their potential strength for development.

From a developmental standpoint, however, there are some limitations in the current data that would need to be addressed through future experimentation. These assays took place under controlled laboratory conditions using a crude release system for the volatiles. For real‐world application the repellent product must last over extended periods, be resilient to environmental factors and perspiration, and be appropriately formulated for application to skin or clothing. This is of particular relevance to natural products, which often require specific formulation to remain effective over several hours.^83^, ^84^ Furthermore, in the *Hyalomma* trials, only PMD was moved to the second‐phase experiments as the best performing commercial repellent. Additional trials conducted over longer periods to assess duration, using both synthetic and naturally derived commercial products for full comparison, and conducted in field or semi‐field settings would prove invaluable. Data generated from these more extensive studies could be used to tailor formulation technologies to enhance the release of the products and ensure their safety and longevity post application.

In summary, blends 3 and 4 show strong repellent effects against multiple tick species, with a performance that surpassed commercial reference products in some assays. Their broad efficacy across genera and orders suggests a wide range of potential applications in vector control. With further optimization, field validation and integration into effective delivery systems, these blends could prove to be highly effective, naturally derived tools for personal protection and integrated vector management strategies.

## 5 CONCLUSIONS

It is concluded that two distinct spruce‐derived blends of the VOCs (+)‐borneol, bornyl acetate, eugenol, isoeugenol and camphor show considerable promise as novel tick‐repellent blends against both *I. ricinus* and *H. excavatum*. In these assays, the two spruce‐derived blends were significantly more effective than the included commercial products, and furthermore were found to work against two tick species that show markedly different questing behaviour. Blend 3, the simpler product because it omits the inclusion of camphor, appears to be marginally more effective overall; however, both blends show near‐equal potential to prevent tick attachment to potential hosts. Given the previously described efficacy of these repellent blends against a broad range of mosquito species, further optimization could lead to the development of a highly effective product with generalized usage against a diverse range of hematophagous pests and vectors of disease.

## CONFLICT OF INTEREST

The authors have no conflicts of interest to declare.

## ETHICS STATEMENT

Full ethical review and approval for rearing of *H*. excavatum ticks *in vivo*, using gerbils and rabbits, was provided and signed for by Aydin Adnan Menderes University veterinary staff and co‐signed by all relevant parties under the SOP ethics code: 64583101/2016/197. For trials assessing the repellent effects of compounds against H. excavatum, assay design was implemented in such a manner as to prevent any contact between ticks and volunteers; prior to assay, however, the full protocol was explained and informed consent obtained for each of the individual volunteers.

## AUTHOR CONTRIBUTIONS

Conceptualization: MJW, KK and TMB. Methodology: MJW, SIY, JCB and TMB. Software: JCB. Validation: MJW, SIY, JCB, SB and TMB. Formal analysis: MJW and JCB. Investigation: SIY, SB, KK and TMB. Resources: KK and TMB. Data curation: MJW, SIY and JCB. Writing – original draft: MJW, SIY, JCB and TMB. Writing – review & editing: MJW, SIY, JCB, SB, KK and TMB. Visualization: MJW, SIY, JCB and TMB. Supervision, MJW, SIY, KK and TMB. Project administration: KK and TMB. Funding acquisition: KK and TMB.

## Data Availability

The data that support the findings of this study are openly available in figshare at https://figshare.com/, reference number 10.6084/m9.figshare.29380829.
